# The relationships of character strengths with job stress, mental wellbeing and perceived stress among financial professionals

**DOI:** 10.3389/fpsyg.2025.1629075

**Published:** 2025-11-07

**Authors:** Talgat Kutebayev, Robert L. Lloyd

**Affiliations:** 1 Department of Psychology, L.N. Gumilyov Eurasian National University, Astana, Kazakhstan; 2 International School of Economics, Maqsut Narikbayev University, Astana, Kazakhstan; 3 Department of Psychology, University of Minnesota, Duluth, MN, United States

**Keywords:** character strengths, job stress, mental wellbeing, perceived stress, financial professionals

## Abstract

**Introduction:**

Character strengths have been related to job stress, mental wellbeing and perceived stress, however, little is known about these associations among financial professionals. The aim of this study was to investigate the relationships between character strengths, job stress, mental wellbeing and perceived stress in this occupational group.

**Methods:**

Data were collected from 523 financial professionals in Kazakhstan using the Job Stress Survey, Values in Action Inventory of Strengths-Virtues 6, Short Warwick-Edinburgh Mental Wellbeing Scale and Perceived Stress Scale.

**Results:**

Regression analysis revealed that interpersonal strengths negatively predicted job stress, while intellectual strengths positively predicted job stress. Results showed that emotional, interpersonal and theological strengths positively predicted mental wellbeing, with theological strengths as the strongest predictor. The study also found that emotional, restraint and theological strengths negatively predicted perceived stress, and emotional strengths were revealed as the strongest predictor.

**Conclusion:**

The findings provide novel insights for organizations to develop preventive interventions based on character strengths for effective stress management, particularly perceived stress, and enhancing mental wellbeing.

## Introduction

According to the International Labor Organization, stress is “the harmful physical and emotional response caused by an imbalance between the perceived demands and the perceived resources and abilities of individuals to cope with those demands” and work-related stress is defined as “the harmful physical and emotional responses that occur when the demands of the job do not match or exceed the capabilities, resources, or needs of the worker, or when the knowledge or abilities of an individual worker or group to cope are not matched with the expectations of the organizational culture of an enterprise” (International Labour Organization, 2016). The financial industry is distinguished by intense and competitive pressure, demanding and challenging work environment, which places significant stress on professionals who frequently experience workplace stressors such as long working hours, tight deadlines, assignment of increased responsibility, inadequate salary, and poor or inadequate support by supervisors or colleagues (Kutebayev et al., 2023; Giorgi et al., 2017; Silva and Navarro, 2012; Giga and Hoel, 2003). Consequently, job stress has become a considerable concern for both employees and financial organizations in particular. It is well known that workplace stress is associated with negative organizational outcomes, including high employee absenteeism and staff turnover, diminished work performance, and poor physical and mental well-being among workers (Schwepker et al., 2021; Vagg and Spielberger, 1999; Caudron, 1998; Karasek and Theorell, 1990; Levi, 1990; Sutherland and Cooper, 1990). The high-pressure financial environment highlights the need to investigate factors that can buffer stress and enhance employees’ mental wellbeing.

In the framework of positive psychology, the study of character strengths has shown that they play a significant role in mitigating job stress and perceived stress, as well as enhancing mental wellbeing (Niemiec, 2023; Casali et al., 2021; Harzer and Ruch, 2015). Character strengths are defined as “positive traits reflected in thoughts, feelings, and behaviors” (Park et al., 2004). The Values in Action (VIA) classification, the model of positive character traits, was proposed by Peterson and Seligman (2004). This classification consists of 24 character strengths, grouped into 6 virtues: (1) intellectual strengths, representing the virtue of wisdom and knowledge, such as creativity, curiosity, judgment, love of learning, and perspective; (2) emotional strengths, that is, the virtue of courage, including the character strengths of bravery, perseverance, honesty, and zest; (3) interpersonal strengths, namely the virtue of humanity, including love, kindness, and social intelligence; (4) civic strengths, which is the virtue of justice, including the character strengths of teamwork, fairness, and leadership; (5) restraint strengths, that is, the virtue of temperance, including forgiveness, humility, prudence, and self-regulation; (6) theological strengths, namely the virtue of transcendence, including the character strengths of appreciation of beauty and excellence, gratitude, hope, humor, and spirituality. In accordance with the VIA classification, the Values in Action Inventory of Strengths (VIA-IS) was developed to measure the character strengths (VIA-IS; Peterson and Seligman, 2004).

According to the Transactional Process (Lazarus and Folkman, 1984) and State–Trait Process (Spielberger et al., 2003) models of occupational stress, individual strengths can influence how employees perceive and appraise workplace stressors. Therefore, examining character strengths in financial professionals is theoretically meaningful, as they may serve as protective resources against job stress and improve mental wellbeing. Character strengths were extensively studied among various occupations such as teachers (Kamboj and Garg, 2021: Darabi et al., 2016: Poormahmood et al., 2017), doctors (Marcisz-Dyla et al., 2022; Kachel et al., 2021; Huber et al., 2020), and nurses (Zhang et al., 2021; Xie et al., 2020; Harzer and Ruch, 2015), but there is a lack of studies investigating the relationships between character strengths, job stress, mental wellbeing and perceived stress among financial professionals. One such study with 601 employees and managers from five large IBEX banks in Spain revealed that theological strength particularly spirituality was significantly associated with reduction of work pressure and work stress, developing transcendent employee behavior and vision, as well as contributing to improve the happiness of bank employees (Robina-Ramírez et al., 2021). Another study with 286 managerial and professional women of large Turkish bank showed that virtues of optimism and proactive behavior were both significantly and positively correlated with psychological well-being, and optimism was a particularly strong predictor of mental well-being (Fiksenbaum et al., 2010). The study with large heterogeneous sample of 974 working adults from different US organizations, such as finance, marketing, and others investigated the impact of employees’ wisdom-related character strengths (i.e., perspective, judgment, originality, curiosity, and love of learning) on stress (Avey et al., 2012). The findings of this study revealed that wisdom strengths were associated with reduced stress among employees.

Although the findings reported in previous studies offer valuable insights, they primarily focused on a limited set of character strengths or virtues, rather than systematically examining how the full set of strengths relates to job stress, mental wellbeing, and perceived stress. This gap indicates the need for a more comprehensive and theoretically grounded examination of these relationships. Therefore, the relationships between character strengths, job stress, mental wellbeing and perceived stress among financial professionals remain insufficiently explored. To our knowledge, no prior research has examined the relationships between character strengths, job stress, job pressure, lack of support, mental wellbeing, and perceived stress among financial professionals. The knowledge of these relationships may help to develop interventions for effective stress management and improve mental health of financial employees, as well as providing insights about relationships specific to particular professional group. In order to fill the existing knowledge gap, the present study aimed to investigate the relationships between character strengths, job stress, job pressure, lack of support, mental wellbeing and perceived stress among financial professionals. Additionally, we explored whether demographic variables such as age, gender, marital status, educational level, work experience and qualification were associated with job stress, job pressure, lack of support, mental wellbeing and perceived stress, contributing to a better understanding of stress and wellbeing in the workplace. To achieve the study aims, we used the Job Stress Survey (JSS; Spielberger, 1991; Spielberger and Vagg, 1999), the Values in Action Inventory of Strengths-Virtues 6 (VIA-IS-V6; McGrath, 2017), the Short Warwick-Edinburgh Mental Wellbeing Scale (SWEMWBS; Stewart-Brown et al., 2009), and the Perceived Stress Scale (PSS-10; Cohen and Williamson, 1988) to collect data on character strengths, job stress, mental wellbeing and perceived stress among Kazakhstani financial professionals working in public and private organizations.

## Methods

### Procedure

A cross-sectional design was used to investigate the relationships between character strengths, job stress, mental wellbeing and perceived stress among financial professionals in Kazakhstan. All participants provided written informed consent before completing the online survey. Participants were informed that the survey was anonymous and confidential. Human Resources departments of the financial organizations distributed the link to the online survey among the employees. The study was approved by the Social Sciences Research Ethics Committee, in accordance with the 1964 Helsinki Declaration (approval number EP_22–27/7).

### Participants

Totally, 523 financial employees were recruited to participate in this study. The sample consisted of 423 women (mean age = 41.4 years; *SD* = 9.8; range: 20–60 years) and 100 men (mean age = 35.8 years; *SD* = 7.9; range: 24–60 years). The mean age of the participants was 40.4 years (*SD* = 9.7; range: 20–60 years) for the total sample. Regarding participants’ age distribution, 19% of participants were between 20 and 30 years (*n* = 100), 34% were between 31 and 40 (*n* = 179), 31% were between 41 and 50 (*n* = 163), and 16% were over 51 (*n* = 81). Most of the participants were married (*n* = 312; 60%), and the rest of participants were unmarried or single (*n* = 116; 22%), widowed, divorced, or separated (*n* = 95; 18%). As for qualification, 40% of the participants were economists (*n* = 210), 30% were financiers (*n* = 156), and 30% were accountants (*n* = 157). In regard to education, most participants had bachelor’s degree (*n* = 449; 86%), and the rest of participants had a postgraduate degree (*n* = 33; 6%) and a vocational degree (*n* = 41; 8%). Concerning work experience, 31% of the participants had 11–20 years of experience (*n* = 163), 30% had 5–10 years (*n* = 154), 20% had less than 5 years (*n* = 106), 19% had more than 20 years (*n* = 100), and the mean duration was 13 years.

### Measures

#### Demographic information

Participants were asked to provide demographic and professional information, including age, gender, marital status (married, single, divorced, separated, widowed), highest educational level (secondary vocational education, bachelor’s degree, master’s degree, doctoral degree, other), duration of work experience, qualification (financier, economist, accountant, other), and organization (government/private).

### Instruments

#### Job stress

The Job Stress Survey (JSS; Spielberger, 1991; Spielberger and Vagg, 1999) was used to assess sources of job-related stress experienced by employees in organization. The JSS is a questionnaire consisting of 30 items explaining 30 different stressful work-related situations (e.g., “frequent interruptions,” “meeting deadlines,” “working overtime”). Employees are asked to rate the perceived severity (intensity) of each stressor event on a 9-point scale (from “1 = low stress” to “9 = high stress”) and frequency of occurrence, i.e., how often the stressor was experienced by the employee during the past year, on a 10-point scale ranging from 0 to 9 + (Vagg and Spielberger, 1999). The severity subscale is formed by average severity of 30 items, and the frequency subscale is formed by average frequency of 30 items. The Job Stress Index is formed by multiplying severity and frequency ratings of all 30 stressor events. In the present study, the total score of Job Stress Index indicated a high level of internal consistency reliability, with a Cronbach’s *α* of = 0.93, the Job Pressure subscale = 0.85 and the Lack of Organizational Support subscale = 0.83.

#### The values in action inventory of strengths-virtues 6 (VIA-IS-V6)

The Values in Action Inventory of Strengths-Virtues 6 (VIA-IS-V6; McGrath, 2017) was used to measure 6 virtues (wisdom, courage, humanity, justice, temperance, transcendence). The VIA-IS-V6 survey consists of 48 items intended to assess an employee’s possession of various character strengths, with 8 items for each virtue, including both positively and negatively keyed statements. Participants responded to each statement (e.g., “I am a brave person,” “I love to learn new things”) using a 5-point Likert scale (from “1 = very much unlike me” to “5 = very much like me”). In this study, the total score of VIA-IS-V6 showed a high level of internal consistency reliability, with a Cronbach’s *α* of = 0.90.

#### The short Warwick-Edinburgh mental wellbeing scale (SWEMWBS)

The Short Warwick-Edinburgh Mental Wellbeing Scale (SWEMWBS; Stewart-Brown et al., 2009) is a 7-item self-report scale that measures mental wellbeing. Participants are asked to answer seven statements (e.g., “I’ve been feeling optimistic about the future,” “I have been thinking clearly”) using a 5-point Likert scale (“None of the time,” “Rarely,” “Some of the time,” “Often” and “All of the time”). In this study, the internal consistency reliability score (Cronbach’s *α*) for the SWEMWBS total score was = 0.85.

#### The perceived stress scale (PSS-10)

The Perceived Stress Scale (PSS-10; Cohen and Williamson, 1988) is a 10-item questionnaire that assesses the degree to which an individual perceives the life as stressful. Participants rated the frequency of experiencing their lives as unpredictable, uncontrollable and overloaded using a five-point Likert scale (from 0 = never to 4 = very often), including both positively worded (e.g., “How often have you felt nervous and stressed?”) and negatively worded (e.g., “How often have you been able to control irritations in your life?”) questions. In this study, the internal consistency reliability score (Cronbach’s α) for the PSS-10 total score was = 0.74.

### Data analyses

Descriptive statistics (frequencies, percentages, means, and standard deviations) were calculated for the demographic variables. Cronbach alpha coefficients were used to assess the validity and internal consistency reliability of all instruments. Multivariate analyses of variance (MANOVAs) were performed to assess statistically significant differences in job stress, mental wellbeing and perceived stress between age groups, work experience durations, gender, marital status, educational level and qualification. *β* coefficient was calculated to determine effect size. Pearson correlation analyses were used to determine positive and negative associations between character strengths with job stress, job pressure, lack of support, mental wellbeing and perceived stress, with statistical significance established at a *p*-value <0.05. Multiple linear regression analyses were carried out to determine if character strengths could predict job stress, mental wellbeing and perceived stress. All identified multivariate outliers were excluded (>3 SD). The assumptions of the multiple linear regressions were tested, including multicollinearity, normality of residuals, homoscedasticity and linearity. The results indicated that none of these assumptions were violated. However, the homoscedasticity assumption of multiple linear regression of character strengths with mental wellbeing was found with violation. To address this, we used multiple linear regression with robust standard errors. The data were analyzed using IBM SPSS version 27.

## Results

Job stress and lack of support were not high for all financial professionals, however, job pressure was high for the overall sample. All financial professionals had moderate mental wellbeing and moderate perceived stress. The top five stressors among all financial professionals were the assignment of increased responsibility, meeting deadlines, inadequate salary, assignment of disagreeable duties and excessive paperwork (see Supplementary Table).

### Correlational and multivariate analyses

The results of the correlation analyses are provided in Table 1. Age and work experience were positively associated with mental well-being and negatively associated with perceived stress, and the correlations were significant. Qualification was significantly and positively associated with mental well-being. Regarding educational level, marital status and gender, no significant associations were found. The results of multivariate analysis of variance revealed a significant difference in job stress, mental wellbeing and perceived stress between age groups *F* (3, 517) = 2.107, *p* = 0.026; Wilks’ Lambda = 0.964, partial η^2^ = 0.012, and also a significant effect of age on mental wellbeing *F* (3, 519) = 4.237, *p* = 0.006, partial η^2^ = 0.024. Games-Howell *post hoc* tests revealed a significant difference between age groups of 20–30 years and 51 + years, so that financial professionals aged 51 + years experienced higher level of mental wellbeing (small effect size). Furthermore, there was a significant difference in job stress, mental wellbeing and perceived stress between work experience durations *F* (3, 517) = 3.389, *p* < 0.001; Wilks’ Lambda = 0.943, partial η^2^ = 0.019, as well as a significant effect of work experience on mental wellbeing *F* (3, 519) = 7.109, *p* < 0.001, partial η^2^ = 0.039. Games-Howell post hoc tests showed significant differences, so that financial professionals with 21 + years of work experience reported high level of mental wellbeing than professionals with less than 5 years, 5–10 years and 11–20 years of experience (small-to-medium effect size). The Box’s M tests for age (*p* = 0.698) and work experience (*p* = 0.381) indicated that there were no assumption violations of homogeneity of variances and covariances matrices. Regarding educational level, qualification, marital status and gender, the multivariate analysis of variance found that there were no significant differences in job stress, mental wellbeing and perceived stress.

**Table 1:** Pearson correlations of gender, age, marital status, work experience, educational level, and qualification with job stress, job pressure, lack of support, mental wellbeing and perceived stress.

Variable	Job stress	Job pressure	Lack of support	Mental wellbeing	Perceived stress
Gender	−0.06	−0.04	−0.05	0.03	−0.05
Age	0.06	0.06	0.07	0.13**	−0.10*
Marital status	−0.07	−0.06	−0.06	−0.08	0.03
Work experience	0.07	0.07	0.05	0.17**	−0.10*
Educational level	0.04	0.03	0.04	0.02	−0.01
Qualification	−0.08	−0.07	−0.08	0.10*	−0.07

*p < 0.05; **p < 0.01; ***p < 0.001.

The results of the correlation analyses are provided in Table 2. Intellectual, emotional, interpersonal, civic, restraint and theological strengths were negatively associated with job stress, job pressure, lack of support and perceived stress, and positively associated with mental wellbeing, and the most correlations were significant. However, the associations of intellectual strengths with job stress, job pressure and lack of support were not statistically significant, as well as the association of restraint strengths with job pressure. Emotional strengths showed the strongest negative association with perceived stress (*r* = −0.46, *p* < 0.001), indicating a moderate relationship. Interpersonal strengths demonstrated the strongest negative associations with job stress, job pressure, and lack of support (*r* = −0.16, −0.14, and −0.14, respectively; *p* < 0.001), representing small but significant correlations. Theological strengths had the strongest positive association with mental wellbeing (*r* = 0.69, *p* < 0.001), reflecting a strong positive correlation.

**Table 2:** Pearson correlations of character strengths with job stress, job pressure, lack of support, mental wellbeing and perceived stress.

Variable	Job stress	Job pressure	Lack of support	Mental wellbeing	Perceived stress
Intellectual strengths	−0.02	−0.02	−0.01	0.50***	−0.28***
Emotional strengths	−0.10*	−0.09*	−0.09*	0.61***	−0.46***
Interpersonal strengths	−0.16***	−0.14***	−0.14***	0.57***	−0.33***
Civic strengths	−0.12**	−0.10**	−0.11**	0.58***	−0.33***
Restraint strengths	−0.10*	−0.07	−0.12**	0.44***	−0.34***
Theological strengths	−0.09*	−0.07*	−0.10*	0.69***	−0.44***

Values in bold are statistically significant at the *p < 0.05; **p < 0.01; ***p < 0.001 levels.

### Multiple linear regression analyses

The results of multiple linear regressions of character strengths with job stress, job pressure and lack of support are provided in Table 3. The overall regression model for job stress was statistically significant (R^2^ = 0.04, *F* (6, 516) = 3.51, *p* = 0.002), explaining 4% of the variance. It was found that intellectual strengths significantly and positively predicted job stress (*β* = 0.16, *p* = 0.014), indicating a small effect size, while interpersonal strengths were a significant negative predictor of job stress (*β* = −0.18, *p* = 0.007), also reflecting a small effect size. However, emotional, civic, restraint and theological strengths did not significantly predict job stress. The regression model for job pressure was statistically significant (R^2^ = 0.03, *F* (6, 516) = 2.69, *p* = 0.014), explaining 3% of the variance. It was revealed that intellectual strengths significantly and positively predicted job pressure (*β* = 0.13, *p* = 0.035), representing a small effect size, whereas interpersonal strengths were a significant negative predictor of job pressure (*β* = −0.17, *p* = 0.012), indicating a small effect size. Regarding emotional, civic, restraint and theological strengths, no significant predictions of job pressure were observed. The results of regression indicated that the model for lack of support was statistically significant (R^2^ = 0.04, *F* (6, 516) = 3.32, *p* = 0.003), explaining 4% of the variance. Intellectual strengths showed to be a significant positive predictor of lack of support (*β* = 0.16, *p* = 0.011), reflecting a small effect size, and interpersonal strengths had a significant negative prediction (*β* = −0.16, *p* = 0.017), representing a small effect size. But, emotional, civic, restraint and theological strengths demonstrated no significant predictions of lack of support.

**Table 3:** Multiple linear regressions of character strengths with JSS scales.

Variable	*R*	*R^2^*	*Adj. R^2^*	*F*	*p*	*β*	*p*	Effect size *(β)*
Job stress	0.20	0.04	0.03	3.51	**0.002**		<0.001	
Intellectual						0.16	**0.014**	small
Emotional						−0.04	0.523	-
Interpersonal						−0.18	**0.007**	small
Civic						−0.05	0.492	-
Restraint						−0.05	0.394	-
Theological						0.01	0.950	-
Job pressure	0.17	0.03	0.02	2.69	**0.014**		<0.001	
Intellectual						0.13	**0.035**	small
Emotional						−0.04	0.485	-
Interpersonal						−0.17	**0.012**	small
Civic						−0.05	0.509	-
Restraint						−0.02	0.757	-
Theological						0.01	0.842	-
Lack of support	0.19	0.04	0.03	3.32	**0.003**		<0.001	
Intellectual						0.16	**0.011**	small
Emotional						−0.01	0.887	-
Interpersonal						−0.16	**0.017**	small
Civic						−0.04	0.624	-
Restraint						−0.08	0.145	-
Theological						−0.03	0.645	-

β, β coefficient effect size; small: β ≥ 0.10; medium: β ≥ 0.30; large: β ≥ 0.50 (Cohen, 1988).Values in bold are statistically significant results.

The results of multiple linear regression of character strengths with mental wellbeing are provided in Table 4. The overall regression model for mental wellbeing was statistically significant (R^2^ = 0.55, F (6, 516) = 106.93, *p* < 0.001), indicating that the six strengths collectively explained 55% of the variance in mental wellbeing. Emotional, interpersonal and theological strengths were significant positive predictors of mental wellbeing, and theological strengths were the strongest positive predictor of mental wellbeing (*β* = 0.39, *p* < 0.001), indicating a medium effect size. Intellectual, civic and restraint strengths showed no significant predictions of mental wellbeing.

**Table 4:** Multiple linear regression of character strengths with mental wellbeing.

Variable	*R*	*R^2^*	*Adj. R^2^*	*F*	*p*	*β*	*p*	Effect size *(β)*
Mental wellbeing	0.75	0.55	0.55	106.93	**<0.001**		0.642	
Intellectual						−0.01	0.835	-
Emotional						0.24	**<0.001**	small
Interpersonal						0.13	**0.004**	small
Civic						0.08	0.100	-
Restraint						0.06	0.129	-
Theological						0.39	**<0.001**	medium

β, β coefficient effect size; small: β ≥ 0.10; medium: β ≥ 0.30; large: β ≥ 0.50 (Cohen, 1988).Values in bold are statistically significant results.

The results of multiple linear regression of character strengths with perceived stress are provided in Table 5. The overall regression model for perceived stress was statistically significant (R^2^ = 0.27, F (6, 516) = 31.29, *p* < 0.001), indicating that the six strengths collectively explained 27% of the variance in perceived stress. It was revealed that emotional, restraint and theological strengths were significant negative predictors of perceived stress, and emotional strengths were the strongest negative predictor of perceived stress (*β* = −0.28, *p* < 0.001), representing a small effect size. In relation to intellectual, interpersonal and civic strengths, there were no significant predictions of perceived stress.

**Table 5:** Multiple linear regression of character strengths with perceived stress.

Variable	*R*	*R^2^*	*Adj. R^2^*	*F*	*p*	*β*	*p*	Effect size *(β)*
Perceived stress	0.52	0.27	0.26	31.29	**<0.001**		<0.001	
Intellectual						0.06	0.359	-
Emotional						−0.28	**<0.001**	small
Interpersonal						−0.03	0.592	-
Civic						0.03	0.649	-
Restraint						−0.10	**0.029**	small
Theological						−0.26	**<0.001**	small

β, β coefficient effect size; small: β ≥ 0.10; medium: β ≥ 0.30; large: β ≥ 0.50 (Cohen, 1988).Values in bold are statistically significant results.

## Discussion

The present study aimed to investigate the relationships between character strengths, job stress, job pressure, lack of support, mental wellbeing and perceived stress among financial professionals, an occupational group that has received insufficient empirical attention. The current study found that age and work experience were positively associated with mental well-being and negatively associated with perceived stress. Additionally, qualification was significantly and positively associated with mental well-being, while gender, marital status and educational level showed no significant associations. Further analysis revealed a significant difference in job stress, mental wellbeing and perceived stress between age groups of 20–30 years and 51 + years, so that financial professionals aged 51 + years experienced higher level of mental wellbeing (Hone et al., 2015; Li et al., 2014; Nilsson et al., 2010; Andrews et al., 1999; Warr, 1992). Moreover, significant differences were found between work experience durations, indicating that financial professionals with 21 + years of work experience reported high level of mental wellbeing than professionals with less than 5 years, 5–10 years and 11–20 years of experience. The differences found between age groups, as well as work experience, may be explained by accumulated experience that older professionals and employees with more than 21 years of experience have gained. These financial professionals might have achieved greater stability, including higher positions, job security, and financial stability, contributing to overall mental wellbeing. Also, they may have established better work-life balance over time, prioritizing their mental wellbeing, and demonstrating increased awareness of the importance of mental health by engaging in practices like regular exercise, mindfulness, and seeking professional help when needed. Regarding gender, marital status, educational level and qualification, there were no significant differences in job stress, mental wellbeing and perceived stress (Jnaneswar and Sulphey, 2021).

The study found that interpersonal strengths were a significant negative predictor of job stress, job pressure and lack of support, while, intellectual strengths, contrary to expectations, significantly and positively predicted job stress, job pressure and lack of support, however, emotional, civic, restraint and theological strengths did not significantly predict job stress, job pressure and lack of support. Therefore, interpersonal strengths showed a negative association with job stress, which was consistent with previous findings (Nappo, 2020; Harzer and Ruch, 2015), as well as with job pressure and lack of support (Harzer and Ruch, 2014). Meanwhile, job stress, job pressure and lack of support decreased as interpersonal strengths increased, and vice versa. Financial professionals with higher interpersonal strengths such as love, kindness, and social intelligence might have positive relationships and communicate effectively, creating a supportive work environment, reducing misunderstandings and conflicts by neutralizing the negative energy or tense emotions that often cause job stress and pressure (Niemiec, 2019; Peterson and Seligman, 2004). The counterintuitive finding that intellectual strengths are associated with higher job stress may be explained through several theoretical perspectives. Intellectual strengths comprise creativity, curiosity, judgment, love of learning, and perspective. Individuals with more intellectual strengths have higher level of education (Ruch et al., 2010). Thus, they may experience increased job stress and job pressure due to their own high expectations, demanding workloads, deadlines, and conflicts with colleagues who may not share their level of intellectual engagement, contributing to a lack of support in the workplace (Solomon et al., 2022). These explanations are consistent with the Demand–Control–Support model, which indicates that job stress arises from demanding work requirements, insufficient workplace social support, and limited decision-making autonomy, restricting employees’ ability to apply their skills effectively (Karasek and Theorell, 1990). Similarly, the Job Demands–Resources model proposes that persistent work demands combined with insufficient resources can result in increased job stress (Demerouti et al., 2001). Additionally, the Effort–Reward Imbalance model explains occupational stress as a discrepancy between the employees’ efforts and the received rewards (Siegrist, 1996; Siegrist et al., 1986).

The results showed that emotional, interpersonal (Korkmaz, 2022; Martínez-Martí et al., 2020; Pressman et al., 2015) and theological strengths (Niemiec, 2023; Ruch and Hofmann, 2017; Kuiper, 2012) were significant positive predictors of mental wellbeing, while intellectual, civic and restraint strengths showed no significant predictions of mental wellbeing. The model explained a large proportion of the variance in mental wellbeing, demonstrating the strong predictive power of these strengths in enhancing the wellbeing of financial professionals. This may be explained that financial professionals with higher emotional, interpersonal and theological strengths are better able to manage their emotions, utilize these strengths effectively, facilitate meaningful connections with others and find purpose (Wagner et al., 2020; Hone et al., 2015; Wood et al., 2011). Theological strengths were identified as the strongest positive predictor of mental wellbeing, which was consistent with previous studies (Jnaneswar and Sulphey, 2021; Azañedo et al., 2021; Martínez-Martí et al., 2020; Pawar, 2016; Karakas, 2010). This may be related to the profound influence of spirituality and faith on employees’ mental health, as a strong belief system provides a sense of meaning and inner peace, which are foundational elements of mental wellbeing (Wagner et al., 2020; Peterson and Seligman, 2004). The lack of significant predictions for intellectual, civic, and restraint strengths may suggest that cognitive, justice and temperance-related strengths are less relevant for maintaining mental wellbeing in high-pressure financial environments. Overall, these findings indicate that mental wellbeing among financial professionals is primarily supported by emotional, interpersonal, and theological strengths, which facilitate goal-directed action, social connectedness, and a sense of purpose, rather than by intellectual, civic, and restraint strengths.

The study also found that emotional, restraint (Martínez-Martí et al., 2020) and theological strengths (Ruch and Hofmann, 2017; Kuiper, 2012) were significant negative predictors of perceived stress. Regarding intellectual, interpersonal and civic strengths, there were no significant predictions of perceived stress. The model explained a large proportion of the variance in perceived stress, indicating the significant role of these strengths in managing stress among financial professionals. This can be explained that financial professionals with higher emotional, restraint and theological strengths regulate their emotions effectively, have positive relationships, demonstrate increased self-control and find meaning (Wagner et al., 2020; Niemiec, 2019; Peterson and Seligman, 2004). Emotional strengths such as bravery, perseverance, honesty, and zest were revealed as the strongest negative predictor of perceived stress. This might be due to the employees’ enhanced resilience, honest and open communication, resulting in reduced levels of perceived stress (Aktan and Khorshid, 2021; Orui and Yasumura, 2019; Sarrionandia et al., 2018). The lack of significant effects for intellectual, interpersonal and civic strengths may indicate that cognitive, relational and justice-related strengths are less directly involved in managing perceived stress among financial professionals. These results suggest that emotional, restraint, and theological strengths are key factors in reducing perceived stress among financial professionals, contributing to goal-oriented action, self-regulation, and a sense of meaning and purpose, while intellectual, interpersonal and justice strengths have a less direct impact.

Regression analysis for job stress indicates that the predictor variables in the model explain only a small proportion of the variance observed in the dependent variable, highlighting the weak explanatory value of character strengths for job stress outcomes compared to the mental wellbeing and perceived stress models. This may suggest that character strengths play a substantial role in reducing perceived stress and enhancing mental well-being, but in case of job stress other factors make a significant contribution such as job demands, workplace relationships, organizational support and culture (Bakker and Demerouti, 2014; Pereira, 2014; Viswesvaran et al., 1999; Cooper et al., 1996). Job stressors are often specific to the work environment and may require different coping strategies than general life stressors. Employees might have developed specific coping strategies to deal with job stressors, which may mitigate the impact of character strengths on work stress. These factors may have a stronger influence on job stress than employees’ character strengths, diminishing their predictive power regarding job stress. Character strengths may play an important role, but their direct applicability to job stressors may be limited in predicting job stress. Future studies may be needed to clarify the predictive power of character strengths to understand the relationships between character strengths and these factors, as it is crucial for developing comprehensive strategies to address job stress.

This study provides valuable insights for organizations to improve mental wellbeing, reduce perceived stress, and may contribute to preventing and mitigating job stress, based on character strengths. Consequently, the direct applicability of character strengths to prevent or mitigate job stress should be considered with prudence. Character strengths are personal traits that can be developed through training (Peterson and Seligman, 2004). The potential of character strengths might still be a significant resource for the financial employees to manage job stress, enhance mental wellbeing, and reduce perceived stress (Niemiec, 2018; McGhee, 2010; Park and Peterson, 2009; Linley and Harrington, 2006). Thus, developing interpersonal strengths in employees, through systematic character strengths interventions and practices integrated into current management and leadership programs, may have modest yet significant effects on reducing job stress, job pressure, and lack of support, but can help employees improve their mental well-being and alleviate perceived stress (Gander et al., 2013). Moreover, developing and using emotional, restraint, and theological strengths can enhance mental well-being and diminish perceived stress, contributing to a positive, productive and health-promoting workplace (Littman-Ovadia and Steger, 2010; Maddi, 2006). In addition, implementing practical positive psychology interventions, such as Mindfulness-Based Strengths Practice (Pang and Ruch, 2019; Niemiec and Lissing, 2016; Ivtzan et al., 2016; Niemiec, 2014) and strengths-based coaching (Elston and Boniwell, 2011), can facilitate an engaging and flourishing work environment (McQuaid and Lawn, 2014). This enables organizations to foster character strengths among employees, resulting in the prevention of job stress, improvement of mental wellbeing, and mitigation of perceived stress.

This study had several limitations. First, the study used a cross-sectional design, which merely assesses existing relationships because data were collected at a single point in time, thus explaining no causal relationships between the variables. Although associations between character strengths, job stress, mental wellbeing, and perceived stress were identified, it is not possible to determine the direction of these relationships or whether a particular variable directly influences another over time. Consequently, any practical applications suggested by these findings should be interpreted carefully, as interventions based on cross-sectional data may not produce the anticipated outcomes. A longitudinal study might give a more comprehensive understanding of the relationships between the study’s variables, clarify their directional effects over time, confirm predictions regarding their relationships, and provide stronger evidence to guide workplace interventions. Second, participants were recruited only from Kazakhstan. This limits the generalizability of the findings, as cultural, economic, and organizational factors may influence these experiences differently. Thus, the results are most relevant for Kazakhstan and similar countries, while their applicability to other nations remains limited. To improve generalizability, future studies are needed, as findings may differ in other countries. Third, the instruments used in this research were self-report surveys, which may slightly increase the possibility of socially desirable responses and thereby limit the robustness of the findings, despite participants being informed that the survey was anonymous and confidential. Future research should employ indirect questioning techniques, neutral item wording, and include social desirability scales. Finally, although participants’ job types were recorded (economists, financiers, accountants), the absence of controls for organizational-level factors, such as hierarchical position and organizational culture, represents a limitation of the study.

In conclusion, this study examined the relationships between character strengths, job stress (including job pressure and lack of support), mental wellbeing and perceived stress among financial professionals. We found that character strengths significantly predicted all three outcomes, with substantially stronger associations for mental well-being and perceived stress than for job stress. Job stress — including job pressure and lack of support — was negatively predicted by interpersonal strengths and positively predicted by intellectual strengths. Mental well-being was positively associated with emotional, interpersonal, and especially theological strengths. Perceived stress was negatively related to emotional, restraint and theological strengths, with emotional strengths showing the strongest relationship. Our findings suggest that character strengths interventions may be more effective in enhancing mental well-being and reducing perceived stress among financial professionals than in alleviating job stress. The abovementioned specific character strengths that showed significant associations with the outcomes in our study may serve as relevant strengths for designing such interventions, as suggested by the study findings. Future studies, exploring the relationships between character strengths, job stress, mental wellbeing and perceived stress among financial professionals in different countries may be required to confirm the findings.

## Data Availability

The raw data supporting the conclusions of this article will be made available by the authors, without undue reservation.
