# Supplementary material for: The relationships of character strengths with job stress, mental wellbeing and perceived stress among financial professionals
Source: Front Psychol. 2025 Nov 7;16:1629075. doi: 10.3389/fpsyg.2025.1629075 (PMC12634367; doi:10.3389/fpsyg.2025.1629075)
Supplement: Supplementary file 1 [file Table_1.DOCX]

**Supplementary Table**

*Descriptive Statistics on* ***Severity, Frequency*** *and I****ndex****Subscales* *for all JSS Items*

| **Total sample** | **Severity** | | **Frequency** | | **Index** | |
| --- | --- | --- | --- | --- | --- | --- |
|  | ***M*** | ***SD*** | ***M*** | ***SD*** | ***M*** | ***SD*** |
| Assignment of disagreeable duties | 3.14 | 2.04 | 2.93 | 1.98 | 12.60 | 15.49 |
| Working overtime | 2.80 | 2.00 | 2.62 | 1.93 | 10.68 | 14.75 |
| Lack of opportunity for advancement | 2.71 | 1.87 | 2.54 | 1.78 | 9.91 | 13.99 |
| Assignment of new or unfamiliar duties | 2.88 | 1.88 | 2.74 | 1.82 | 10.88 | 13.85 |
| Fellow workers not doing their jobs | 2.67 | 1.90 | 2.48 | 1.78 | 9.55 | 13.81 |
| Inadequate support by supervisor | 2.55 | 1.84 | 2.39 | 1.73 | 8.85 | 12.56 |
| Dealing with crisis situations | 2.87 | 1.88 | 2.62 | 1.68 | 10.20 | 12.83 |
| Lack of recognition for good work | 2.58 | 1.85 | 2.48 | 1.80 | 9.32 | 13.38 |
| Performing tasks not in job description | 2.65 | 1.86 | 2.44 | 1.70 | 9.18 | 12.12 |
| Inadequate or poor quality equipment | 2.59 | 1.98 | 2.47 | 1.92 | 9.74 | 15.23 |
| Assignment of increased responsibility | 3.79 | 2.41 | 3.57 | 2.38 | 18.78 | 21.76 |
| Periods of inactivity | 2.15 | 1.57 | 2.02 | 1.49 | 6.42 | 9.48 |
| Difficulty getting along with supervisor | 2.26 | 1.73 | 2.05 | 1.54 | 6.89 | 10.84 |
| Experiencing negative attitudes toward the organization | 1.96 | 1.45 | 1.85 | 1.36 | 5.37 | 8.33 |
| Insufficient personnel to adequately handle an assignment | 2.37 | 1.70 | 2.21 | 1.56 | 7.53 | 10.43 |
| Making critical on-the-spot decisions | 2.90 | 1.92 | 2.70 | 1.82 | 10.92 | 14.13 |
| Personal insult from customer/consumer/colleague | 2.14 | 1.69 | 1.89 | 1.38 | 5.87 | 9.31 |
| Lack of participation in policy-making decisions | 2.22 | 1.58 | 2.05 | 1.47 | 6.67 | 10.07 |
| Inadequate salary | 3.00 | 2.32 | 2.81 | 2.30 | 13.28 | 20.37 |
| Competition for advancement | 2.15 | 1.67 | 2.06 | 1.62 | 6.92 | 12.10 |
| Poor or inadequate supervision | 2.11 | 1.66 | 1.95 | 1.52 | 6.25 | 10.33 |
| Noisy work area | 2.19 | 1.70 | 2.04 | 1.63 | 6.88 | 12.05 |
| Frequent interruptions | 2.49 | 1.79 | 2.37 | 1.76 | 8.71 | 12.59 |
| Frequent changes from boring to demanding duties | 2.36 | 1.67 | 2.22 | 1.62 | 7.61 | 11.20 |
| Excessive paperwork | 2.95 | 2.03 | 2.81 | 2.00 | 12.03 | 16.35 |
| Meeting deadlines | 3.52 | 2.31 | 3.32 | 2.27 | 16.37 | 20.30 |
| Insufficient personal time | 2.66 | 2.13 | 2.56 | 2.11 | 10.88 | 17.67 |
| Covering work for another employee | 2.74 | 1.97 | 2.55 | 1.85 | 10.10 | 13.90 |
| Poorly motivated coworkers | 2.70 | 1.97 | 2.50 | 1.86 | 10.08 | 14.68 |
| Conflicts with other department | 2.12 | 1.66 | 1.98 | 1.53 | 6.45 | 10.91 |
